# Transarterial chemoembolization in hepatocellular carcinoma: exploring its role in vascular invasion and extrahepatic metastasis: A systematic review

**DOI:** 10.1097/MD.0000000000041570

**Published:** 2025-02-21

**Authors:** Muhammad Usman Younas, Abdullah Saeed, Muhammad Ramzan, Muhammad Junaid Tahir, Khabab Abbasher Hussien Mohamed Ahmed, Ali Ahmed

**Affiliations:** a Pakistan Kidney and Liver Institute and Research Center, Lahore, Pakistan; b Shaukat Khanum Memorial Cancer Hospital and Research Centre, Lahore, Pakistan; c Faculty of Medicine, University of Khartoum, Khartoum, Sudan; d Division of Infectious Diseases and Global Public Health, School of Medicine, University of California San Diego, La Jolla, CA.

**Keywords:** chemoembolization, extrahepatic metastasis, hepatocellular carcinoma, liver cancer, vascular invasion

## Abstract

**Background and aims:**

Transarterial chemoembolization (TACE) is a significant intervention in hepatocellular carcinoma (HCC) management, but controversies persist regarding its application in advanced cases with vascular invasion or extrahepatic metastasis. This systematic review aims to explore TACE’s efficacy and safety in these cases.

**Methods:**

A literature search was conducted on TACE in HCC patients with vascular invasion or extrahepatic metastasis. The study compared TACE with surgical resection/chemotherapeutic drugs or with no group as well. Safety was assessed for adverse outcomes and efficacy, including overall survival, mean survival, and progression-free survival (PFS). Data extraction included study characteristics, patient demographics, intervention details, outcomes, and adverse events.

**Results:**

A study of 28 studies involving 3740 patients found that TACE showed diverse safety and efficacy outcomes. Safety evaluations focused on liver function tests, while patient-reported symptoms included fever, pain, vomiting, and gastrointestinal issues. Overall survival was under 10 months in 9 studies, with PFS lower in the TACE group compared to conservative treatments. Survival rates ranged from 93.4% at 3 months to 13% at 24 months across studies. The study identified potential subsets where TACE exhibited efficacy, especially in cases with favorable liver function or specific tumor classifications.

**Conclusion:**

Our findings suggest a potential role for TACE in certain subsets of advanced HCC patients. Tailored treatment algorithms, informed by rigorous clinical trials and considering various prognostic factors, hold the potential to enhance the management and outcomes for this complex patient population.

## 1. Introduction

Hepatocellular carcinoma (HCC) stands as the primary form of liver cancer, originating from hepatocytes, constituting approximately 75% to 85% of all liver cancers globally.^[1]^ Its prevalence has witnessed a steady rise, with estimates suggesting over a million new cases annually.^[2]^ The mortality rates are alarmingly high, making it the third leading cause of cancer-related deaths worldwide with a 5-year survival rate of only 18%.^[1]^ Previously, HCC was largely attributed to hepatitis B (HBV) and Hepatitis C (HCV) viruses. However, the pivotal discovery and implementation of the HBV vaccine marked a significant milestone in HCC prevention, showcasing a 75% reduction in its incidence among vaccinated populations.^[3]^ Moreover, the landscape of HCC is evolving, with a shift towards nonalcoholic fatty liver disease (NAFLD) emerging as a predominant cause in certain regions.^[3]^ This highlights a growing concern in the Western world, highlighting the necessity for tailored preventive strategies amid changing disease patterns.^[3]^

Given HCC malignant growth, multiple guidelines have been devised to treat and manage it.^[4–8]^ The current guidelines for HCC include the Asian Clinical Practice Guidelines for the Management of Hepatocellular Carcinoma, the American Association for the Management of Liver Disease guidelines, the European Society for Medical Oncology (ESMO) Clinical Guideline, and The American Gastroenterological Association Guideline etc.^[4–8]^ For staging the Barcelona Clinic Liver Cancer (BCLC) staging system and Child–Pugh Scoring System (CPS), delineate patients into several stages based on tumor size, nodal involvement, metastasis, and liver function.^[9]^ This stratification aids in tailoring treatment approaches for patients at distinct disease stages.

Treatment modalities for HCC encompass a spectrum ranging from surgical interventions such as resection or transplantation to nonsurgical options.^[6,10,11]^ Among these, transarterial chemoembolization (TACE) stands as a vital intervention. TACE involves the intra-arterial administration of chemotherapeutic agents, often combined with embolic agents, directly into the tumor-feeding arteries.^[12]^

TACE finds application primarily in patients with preserved liver function, typically CPS class A, and sometimes in class B or C patients with localized disease.^[13]^ The Child–Pugh score (CPS) categorizes liver function into Class A, B, and C, providing a crucial framework to assess liver disease severity.^[13]^ Class A indicates well-preserved liver function. Class B represents moderate liver dysfunction, indicating a more significant compromise in liver function compared to Class A. Class C reflects severe liver dysfunction, posing significant challenges in managing the disease. Patients in this category have decompensated cirrhosis, experiencing substantial symptoms and complications. Treatment decisions in these cases need careful consideration due to the heightened risks associated with compromised liver function.^[13]^

Guidelines, notably the ESMO Clinical Practice Guidelines, recommend TACE as a treatment adjunct for CPS class A patients with resectable tumors and CPS class B/C when the disease is still localized within the liver or if portal vein thrombosis occurs.^[6]^ However, the role of TACE in cases involving vascular invasion or extrahepatic metastasis remains an area of exploration and debate.^[14,15]^ This is attributed to the concerns regarding potential complications post-procedure, such as acute liver failure or intrahepatic tumor progression.^[9]^

Recent reviews have emphasized the necessity to delineate the significance of Child–Pugh classes in TACE utilization.^[14,15]^ These studies highlight the need for a more comprehensive understanding of how this classification system influences treatment outcomes. While class A patients often demonstrate favorable responses to TACE, the study^[13]^ illuminated the lack of detailed elucidation regarding the inclusion of class B or C patients, whose compromised liver function might pose challenges and demand cautious consideration during TACE application. This gap in prior investigations highlights the crucial role of delineating the nuances of Child–Pugh classification in the context of TACE for HCC management. This review aims to systematically assess the efficacy and safety of TACE in addressing HCC with vascular invasion or extrahepatic metastasis. By assimilating data from a comprehensive array of studies, this review intends to shed light on the potential benefits, limitations, and varying outcomes associated with TACE in this specific cohort of patients.

## 2. Methods

The protocol of this systematic review was registered on PROSPERO (CRD42023477066). The reporting of this review followed the Preferred Reporting Items for Systematic Reviews and Meta-Analyses (PRISMA).^[16]^

## 3. Literature search strategy

A literature search was conducted across multiple electronic databases, including PubMed/MEDLINE, Cochrane Library, and Scopus. The search was performed from October 30, 2023, utilizing relevant keywords and medical subject headings (MeSH) terms. The following search terms were used during the process: “hepatocellular carcinoma,” “HCC,” “vascular invasion,” “extrahepatic metastasis,” “Transarterial chemoembolization,” and “TACE” Additionally, manual searches of reference lists from relevant articles were conducted to identify additional studies for potential inclusion. Details of the keywords incorporated are provided in Table S1, Supplemental Digital Content, http://links.lww.com/MD/O391.

## 4. Inclusion and exclusion criteria

The eligibility criteria followed the Population, Intervention, Comparison, Outcomes and Study strategy:

Population: HCC patients with vascular invasion or extrahepatic metastasis and classified as CPS A or B.

Intervention: TACE alone.

Comparators: Surgical resection/chemotherapeutic drugs/no group.

Outcome: Safety is defined by adverse outcomes, as defined by common terminology criteria for adverse events (CTCAE), version 5.0,^[17]^ as well as mortality and efficacy as overall survival, mean survival, and progression-free survival (PFS) (the duration during and after a medical intervention, where cancer does not worsen or progress) at 3, 6, 12, and 24 months.

Study design: Randomized controlled trials and observational studies.

Exclusion criteria comprised studies not available in English, conference abstracts, case reports, editorials, letters, and studies lacking adequate information on outcomes related to TACE in HCC patients with vascular invasion or extrahepatic metastasis.

## 5. Study selection process

Two independent reviewers (MUY and AS) screened the titles and abstracts of retrieved articles to assess their eligibility based on the predetermined inclusion and exclusion criteria. Full-text articles of potentially relevant studies were retrieved and further evaluated for final inclusion. Any disagreements between the reviewers were resolved through discussion and consensus, involving a third reviewer (MZ) when necessary.

## 6. Data extraction

A predeveloped data extraction form was used to systematically extract pertinent information from the included studies. Data extracted included study characteristics (author[s], publication year, study design), patient demographics (age, gender distribution), intervention details (e.g., types of TACE, chemotherapeutic agents used), outcomes (e.g., overall survival, PFS, adverse events), and any other relevant findings.

## 7. Quality assessment

The quality and risk of bias assessment of included studies were evaluated using the Newcastle-Ottawa scale (NOS).^[18]^ Studies were assessed for methodological rigour, potential sources of bias, and the overall quality of evidence presented.

## 8. Data synthesis and analysis

A narrative synthesis approach was employed to summarize the findings from the included studies. Due to heterogeneity among the studies in terms of methodologies and outcomes, a meta-analysis was not feasible. Instead, results were qualitatively synthesized to provide a comprehensive overview of the evidence related to TACE in HCC patients with vascular invasion or extrahepatic metastasis.

## 9. Ethics statement

Ethical approval was not required because this is a systematic review.

## 10. Results

### 10.1. Literature search

**Figure 1.**
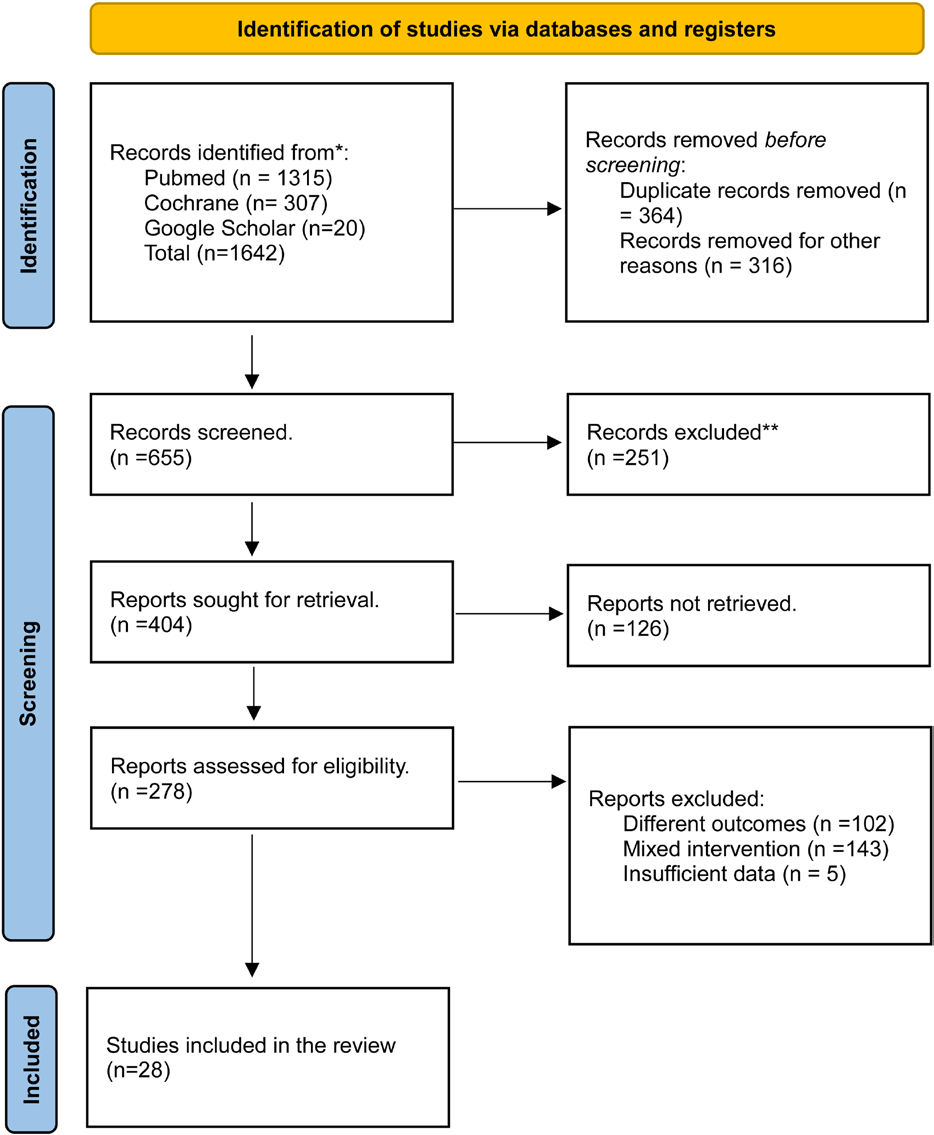
Preferred reporting items for systematic reviews and meta-analyses (PRISMA) flow chart of literature search.

After employing the relevant search string, a total of 1622 articles were retrieved. Removal of duplicates led to 1278 articles, of which 655 were deemed eligible for in-depth review after a thorough screening of titles and abstracts. Ultimately, twenty-eight studies were included in the final qualitative synthesis of data.^[19–46]^ Figure 1 summarizes our selection process for the systematic review.

## 11. Included studies’ characteristics

**Figure 2.**
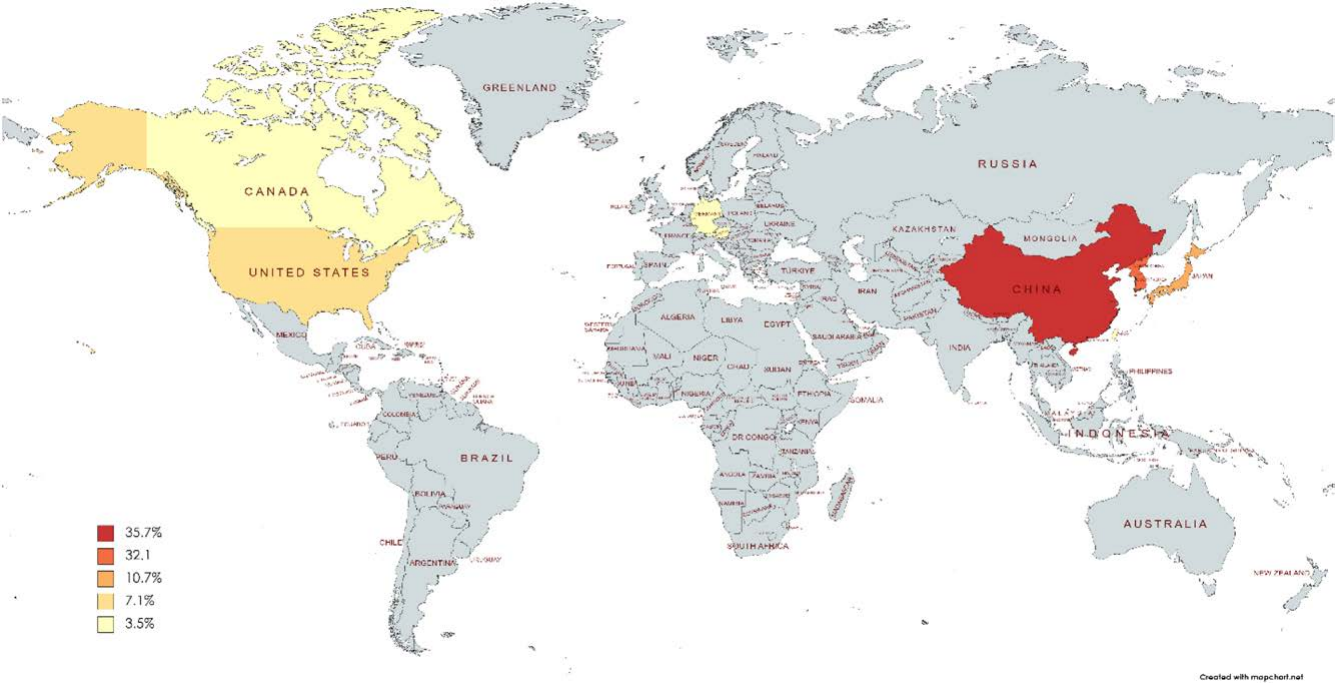
Demographics of included study.

A total of twenty-eight studies with data from 3740 patients are included in the review. Twenty of them were retrospective cohort in nature,^[19,21,22,24–26,28–31,33–35,37–39,41–43,45,46]^ while 9 were prospective.^[20,23,27,32,36,38,40,44,45]^ Of them, 10 were from China,^[32,33,35,36,38–41,43,45]^ 9 were from Korea,^[19,20,24,25,27,29–31,44]^ 3 from the United States Of America,^[22,26,28]^ 2 from Japan,^[21,46]^ and 1 from Germany,^[42]^ Canada,^[23]^ Austria,^[34]^ and Taiwan.^[37]^ Figure 2 illustrates the different regions of our participants. 85.1% of the included patients were male while 14.8% were females. The median age of the cohort was 55.1 years. 1974 patients were class A CPS, while 581 were class B and only 4 studies had a total of 11 patients from class C. Two studies did not report the CPS class.^[38,46]^ Detailed characteristics of our included demographics are tabulated in Table 1.

**Table 1:** Baseline characteristics of included studies.

Author, yr	Country	Design	Intervention	Control	Follow-up	Population	Male, %	Median age	CPS (A/B/C) %	Agent used	Vascular invasion, %	Extrahepatic metastasis, %
Chung et al, 1995	Korea	Retrospective	TACE	N/A	36 mo	110	90	N/A	85/15/0	Doxorubicin, mitomycin	100	N/A
Lee et al, 1997	Korea	Prospective	TACE	N/A	N/A	31	84	52	100/0/0	Doxorubicin	100	0
Uraki et al, 2004	Japan	Retrospective	TACE	N/A	15 mo	61	85	63	74/26/0	Doxorubicin	100	N/A
Geordiages et al, 2005	USA	Retrospective	TACE	N/A	18 mo	32	91	65	72/28/0	Cisplatin, doxorubicin and mitomycin C	100	0
Molinari et al, 2006	Canada	Prospective	TACE	N/A	N/A	47	87.2	63	47/0/0	Doxorubicin, lipiodol	100	N/A
Kim et al, 2009	Korea	Retrospective	TACE	TACI	24 mo	49	90	54	61/35/4	Cisplatin, doxorubicin	100	N/A
Kim et al, 2009	Korea	Retrospective	TACE	N/A	N/A	149	87	52	71/28/1	Cisplatin	100	29
Carr et al, 2010	USA	Retrospective	TACE	N/A	N/A	90	76	57	72/28/0	Cisplatin	100	0
Kim et al, 2010	Korea	Prospective	TACE	HAIC	N/A	31	N/A	55	65/36/0	Doxorubicin	N/A	0
Lewandowski et al, 2010	USA	Retrospective	TACE	N/A	24 mo	23	N/A	N/A	74/26/0	Doxorubicin, cisplatin, mitomycin	N/A	N/A
Yoo et al, 2011	Korea	Retrospective	TACE	systemic chemotherapy (s-chemo)	24 mo	154	84	52	59/41/0	Cisplatin or doxorubicin	79	100
Chung et al, 2011	Korea	Retrospective	TACE	Supportive care	24 mo	125	89	54	69/31/0	Doxorubicin, mitomycin	100	N/A
Luo et al, 2011	China	Prospective	TACE	N/A	N/A	84	98	44	N/A	Lobaplatin, epirubicin, mitomycin C and iohexol	100	9.5
Zhou et al, 2011	China	Retrospective	TACE	Liver transplantation/hepatectomy plus thrombectomy/hepatectomy plus thrombectomy combined with adjuvant chemotherapy and conservative treatment	N/A	10	100	N/A	4/6/0	5-fluorouracil, adriamycin	100	N/A
Pinter et al, 2012	Austria	Retrospective	TACE	Sorafenib	N/A	97	82	N/A	59/41/0	Doxorubicin	35	41
Peng et al, 2012	China	Retrospective	TACE	HR	5 yr	402	93	55	97/3/0	Carboplatin, epirubicin, mitomycin C	100	0
Niu et al, 2012	China	Prospective	TACE	Conservative treatment	24 mo	115	93	45	77/23/0	Cisplatin, epirubicin, iohexol	100	0
Liu et al, 2014	Taiwan	Retrospective	TACE	HR	5 yr	181	34.2	64	70/25/4	Adriamycin, Lipiodol	100	N/A
Ye et al, 2014	China	Retrospective	TACE	HR	3 yr	86	93	45	78/8/0	Doxorubicin, lipiodol, cisplatin	100	N/A
Wu et al, 2015	China	Prospective	TACE	N/A	24 mo	48	83.3	N/A	84/14/0	Adriamycin, Lipiodol	N/A	100
Zheng et al, 2016	China	Retrospective	TACE	HR	5 yr	134	73.1	51	101/33/0	cisplatin, mitomycin, epirubicin, lipiodol	100	N/A
Lee et al, 2016	Korea	Retrospective	TACE	HR Sorafenib	24 mo	80	83.75	58	58/22/0	Doxorubicin	100	N/A
Ye et al, 2016	China	Prospective	TACE	Surgical intervention sorafenib palliative treatment	24 mo	274	85	48	N/A	Doxorubicin, lipiodol, cisplatinum	100	N/A
Bettinger et al, 2017	Germany	Retrospective	TACE	TACE + sorafenib	N/A	42	85.7	68	33/9/0	Epirubicin, mitomycin, lipiodol	N/A	100
Xiang et al, 2019	China	Retrospective	TACE	Conservative treatment	N/A	675	91.4	51	265/85/0	Lipiodol, 5-fluorouracil, adriamycin	100	N/A
Kim et al, 2019	Korea	Prospective	TACE	Sorafenib	94.6 mo	117	85.5	57	100/15/2	Doxorubicin, lipiodol	N/A	100
Hu et al, 2020	China	Prospective	TACE	HAIC	24 mo	24	83.3	54	21/3/0	Epirubicin, lipiodol	100	N/A
Hori et al, 2020	Japan	Retrospective	TACE	N/A	5 yr	14	85.7	61	N/A	cisplatin, fluorouracil, epirubicin, mitomycin C	N/A	100

CPS = Child–Pugh score, HAIC = hepatic arterial infusion chemotherapy, HR = hepatic resection, N/A = not available, TACE = transarterial chemoembolization, USA = United States of America.

## 12. Risk of bias assessment

In analyzing the studies included in this review, most of our studies were adequately reported, had a high score and thus carried a low risk of bias. it was observed that eleven of them were attributed with a NOS score of 7.^[24,27,28,32,34,36,37,41–43,45]^ This score signifies a methodological strength characterized by well-designed research, encompassing meticulous control for biases in the selection of study groups, the establishment of comparability between cohorts, and dependable ascertainment of the outcome of interest.

Only 2 of the studies were scored as 4, attributing to a higher risk of bias.^[21,38]^ These findings suggest potential areas where these particular studies might have encountered methodological weaknesses or limitations. A detailed tabulation of ROB has been pasted as Table 2.

**Table 2:** Risk of bias of included studies.

Study	Selection				Comparability	Outcome			Total
	1	2	3	4	5	6	7	8	
Chung et al, 1995	★	★	–	–	–	★	★	★	5
Lee et al, 1997	–	–	★	★	–	★	★	★	5
Uraki et al, 2004	★	★	★	–	–	–	–	★	4
Geordiages et al, 2005	–	★	–	★	–	★	★	★	6
Molinari et al, 2006	–	–	–	★	–	★	★	★	5
Kim et al, 2009	★	–	★	★	★	★	★	★	7
Kim et al, 2009	★	★	★	★	–	★	–	★	6
Carr et al, 2010	★	★	–	★	–	★	★	★	6
Kim et al, 2010	★	★	★	★	–	★	★	★	7
Lewandowski et al, 2010	★	★	★	★	–	★	★	★	7
Yoo et al, 2011	★	★	–	★	–	★	★	★	5
Chung et al, 2011	–	–	★	★	–	★	★	★	5
Luo et al, 2011	★	–	★	★	★	★	★	★	7
Zhou et al, 2011	★	★	–	★	–	★	★	★	6
Pinter et al, 2012	★	★	★	★	–	★	★	★	7
Peng et al, 2012	★	★	–	★	–	★	★	★	6
Niu et al, 2012	★	★	★	★	–	★	★	★	7
Liu et al, 2014	★	★	–	★	–	★	★	★	7
Ye et al, 2014	–	–	★	★	–	–	★	★	4
Wu et al, 2015	–	–	★	★	–	★	★	★	5
Zheng et al, 2016	★	–	★	★	★	★	★	★	7
Lee et al, 2016	★	★	–	★	–	★	★	★	6
Ye et al, 2016	–	–	–	★	–	★	★	★	5
Bettinger et al, 2017	★	–	★	★	★	★	★	★	7
Xiang et al, 2019	★	★	★	★	–	★	★	★	7
Kim et al, 2019	–	–	★	★	–	★	★	★	5
Hu et al, 2020	★	–	★	★	★	★	★	★	7
Hori et al, 2020	★	★	–	★	–	★	★	★	6

Star represents if the studied fulfilled the criteria according to NOS.

NOS = the Newcastle-Ottawa scale.

## 13. Safety

Of our included studies, 19^[19–25,27,31,32,34,35,38,41,43–46]^ reported regarding the safety of TACE as adverse events caused by the intervention as defined by CTCAE, version 5.0. 1-month mortality is not typically graded in CTCAE, however, was reported due to its important connection with the paper. The studies measured liver function via liver function tests, especially aspartate transaminase (AST), alanine aminotransferase (ALT) and bilirubin profile. At the same time, the rest of the symptomatology was patient-reported.

Of them, 9 reported 1-month mortality^[19,20,22,25,32,34,35,43,45]^ with the highest 1 reported by Xiang et al, with eighteen patients succumbing to death in a month.^[43]^ Fever was reported in 4 studies,^[20,35,38,45]^ pain was reported in 3 studies,^[20,35,46]^ while vomiting and gastrointestinal dysfunction such as diarrhea or constipation were reported in 3^[35,38,46]^ and 5 studies^[19,27,31,34,45]^ respectively. A detailed adverse event profile has been tabulated in Table 3.

**Table 3:** Adverse reactions after TACE.

Complications	1-mo mortality	Fever’	Pain	Vomiting	Ascites	Liver function deteriorated	Gastrointestinal toxicities	Pleura effusion	Liver failure	Cholecystitis	Paralytic ileus	Hyperbilirubinemia	Anemia	Neutropenia	SBP or sepsis	Thrombocytopenia	ARF
Chung et al, 1995	14	N/A	N/A	N/A	0	7	0	0	2	0	0	N/A	0	N/A	0	N/A	N/A
Lee et al, 1997	0	65	52	N/A	6	N/A	N/A	6	0	0	3	10	0	N/A	0	N/A	N/A
Uraki et al, 2004	N/A	N/A	N/A	N/A	N/A	N/A	N/A	N/A	1	N/A	N/A	N/A					N/A
Georgiades et al, 2005	0	N/A	N/A	N/A	0	N/A	N/A	0	0	0	0	N/A	0	N/A	0	N/A	N/A
Molinari et al, 2006	N/A	N/A	N/A	N/A	5	5	N/A	N/A	N/A	N/A	N/A	N/A	N/A	1	1	N/A	N/A
Kim et al, 2009	N/A	N/A	N/A	N/A	0	1	N/A	N/A	N/A	N/A	N/A	N/A	N/A	N/A	N/A	N/A	2
Kim et al, 2009	1	N/A	N/A	N/A	N/A	N/A	N/A	N/A	N/A	N/A	N/A	N/A	N/A	N/A	N/A	N/A	N/A
Kim et al, 2010	N/A	N/A	N/A	N/A	0	N/A	23	0	0	0	0	39	3	6	0	N/A	N/A
Lewandowski et al, 2010	N/A	N/A	N/A	N/A	N/A	36	N/A	N/A	N/A	N/A	N/A	N/A	N/A	N/A	N/A	N/A	N/A
Luo et al, 2011	1	N/A	N/A	N/A	0	35	N/A	0	0	2	0	N/A	2	N/A	0	N/A	N/A
Chung et al, 2011	0	N/A	N/A	N/A	12	N/A	6	0	0	0	5	N/A	0	N/A	5	N/A	N/A
Pinter et al, 2012	N/A	N/A	N/A	N/A	50	15	12	9	0	0	0	N/A	0	9	9	N/A	N/A
Peng et al, 2012	0	52	56	49	0	N/A	N/A	1	2	2	0	N/A	0	N/A	0	N/A	N/A
Hu et al, 2020	1	4	N/A	N/A	0	4	1	N/A	N/A	N/A	N/A	2	0	1	N/A	2	N/A
Zheng et al, 2016	N/A	N/A	N/A	N/A	1	N/A	N/A	2	N/A	N/A	N/A	N/A	N/A	N/A	N/A	N/A	N/A
Ye HH et al, 2016	N/A	62	119	49	0	N/A	N/A	1	1	N/A	N/A	N/A	N/A	N/A	N/A	N/A	N/A
Xiang et al, 2019	18	N/A	N/A	N/A	N/A	N/A	N/A	N/A	3	N/A	N/A	9	N/A	N/A	N/A	N/A	N/A
Kim et al, 2019	N/A	N/A	N/A	N/A	N/A	N/A	N/A	N/A	N/A	N/A	N/A	N/A	N/A	N/A	N/A	N/A	N/A
Hori et al, 2020	N/A	N/A	3	3	N/A	N/A	N/A	N/A	N/A	N/A	N/A	N/A	1	1	N/A	N/A	N/A

ARF = acute renal failure, N/A = not available, SBP = spontaneous bacterial peritonitis, TACE = transarterial chemoembolization.

## 14. Efficacy

Ten of the total studies reported effectiveness of TACE for metastatic HCC.^[20,30–32,36,38,41,44–46]^ Yoo et al, reported all outcomes, categorizing them as patients with stage 1 tumor (tumor confined to the liver) and CPS class A or B, Patients with stage 3 tumor (tumor has invaded the nearby vessels) with CPS class A and lastly patients with tumor stage 3, CPS class B.^[30]^

Of them, 9 reported overall survival in months,^[20,30–32,36,38,44–46]^ with the highest survival months recorded by Yoo et al, as 12.3 months for TACE alone.^[30]^ The study by Hu et al demonstrated contrasting results.^[45]^ Herein the TACE group had a median survival of only 4 months, compared to chemotherapy which was estimated at around 20.8 months. In all studies reporting overall survival (as measured by mean survival time of all included patients), the average survival was less than or equal to ten months, except for Yoo et al, whereas mentioned earlier, it was 12.3 months.^[30]^ However, that survival rate was only observed in Tumor stage 1, CPS class A or B, unlike T3, CPA where the mean survival was only 5 months and T3, CPS B, where 2.6 months were recorded as the overall survival (OS).

Progression-free survival was reported by 1 study,^[45]^ where it again was lesser than conservative treatment and recorded to be 1.5 months, compared to 9.6 in the other group. Survival at 3 months was reported by 3 studies,^[31,32,38]^ of which the highest was 93.4% in the study by Ye HH and colleagues.^[38]^

When survival for 6 months was evaluated, 5 studies were found to be reporting the outcome.^[20,30–32,38]^ Of them the highest recorded was 86.7% in the study Ye et al.^[38]^ Yoo et al, showed a close margin with 84% of their study participants surviving for 6 months,^[30]^ however, the result was only for tumor staging 1, CPS class A or B. Mean survival at 12, 18 and 24 months were reported in 6,^[20,31,32,38,41,46]^ 3^[30,31,41]^ and 3 studies^[30,32,38]^ respectively. The highest survival at 12 months was 77.6, while that at 18 and 24 months were 79.1% and 13%, respectively. For 24 months, tumor class 1 and CPS category A or B were found to be leading.

Chung and colleagues emphasized the pivotal role of parenchymal tumor extent in predicting both therapy efficacy and complications.^[19]^ Their findings revealed a significant contrast in survival times: patients with limited parenchymal tumors (confined to 1 or 2 segments of a hepatic lobe) survived for 22 months, notably longer than those with more extensive tumors, surviving for just 5 months.^[19]^ Lee’s research highlighted the impact of tumor type on survival, noting that patients with nodular-type HCC had notably extended survival compared to those with diffuse-type HCC.^[20]^ Uraki et al identified several significant variables linked to prognosis, including tumor response, ascites, accumulation of iodized oil in tumor thrombi, Okuda classification, and tumor size. These factors were found to independently predict patient outcomes, as highlighted in their multivariate analysis.^[21]^

In examining the efficacy of TACE in the context of CPSs, the studies underscored the pivotal role CPS plays in determining outcomes. Yoo et al revealed substantial differences in median survival times based on CPS classifications.^[30]^ They reported a notable disparity in survival rates between patients classified as Child–Pugh A and those classified as Child–Pugh B in the context of T3-stage HCC. Patients classified as Child–Pugh A exhibited median survival times of 10 months with TACE/TACI (transarterial chemo-infusion) with s-chemo and 7.1 months with TACE/TACI alone. However, those classified as Child–Pugh B displayed considerably shorter median survival times, recording 5 months with TACE/TACI with s-chemo and merely 2.6 months with TACE/TACI alone.^[30]^ Moreover, Kim et al’s study in 2009 highlighted the significant impact of CPS on survival rates. They found that TACE demonstrated notable survival benefits compared to conservative management, particularly in patients classified as Child–Pugh A, where the median survival period was 14.9 months compared to 4.4 months in the TACI group. In Child–Pugh B categorized patients, the difference was even more pronounced, with survival rates of 5.5 months in the TACE group compared to 1.3 months in the TACI group.^[24]^ Chung’s research in 2011 further emphasized this disparity, showing that treatment with TACE correlated with notably enhanced survival periods in both Child–Pugh class A and B categories, further accentuating the differential impact of CPS on treatment outcomes.^[31]^ A detailed efficacy report of each study is demonstrated in Table 4.

**Table 4:** Efficacy of TACE in included studies.

Author, yr	OS (mo) TACE/other treatment	PFS (mo)	Survival (%) at 3 mo	Survival (%) 6 mo	Survival (%) at 12 mo	Survival (%) at 18 mo	Survival (%) at 24 mo
Lee et al, 1997	5/3		N/A	74	36	N/A	N/A
Yoo et al, 2011	T1 CP A/B = 12.5/6.9	N/A	N/A	T1 CP A/B = 84	T1 CP A/B = 58	T1 CP A/B = 24	T1 CP A/B = 13
	T3, CP A = 5/2.9			T3, CP A = 41	T3, CP A = 15	T3, CP A = 7	T3, CP A = 3
	T3, CP B = 2.6/1.6			T3, CP B = 18	T3, CP B = 5	T3, CP B = 0	T3, CP B = 0
Chung et al, 2011	5.6/2.2	N/A	46	25	17	11	N/A
Luo et al, 2011	7.1/1.4	N/A	86	56	31	N/A	9
Niu et al, 2012	8.7/1.4	N/A	N/A	N/A	N/A	N/A	N/A
Zheng et al, 2016	N/A	N/A	N/A	N/A	77.6	79.1	N/A
Ye HH et al, 2016	10.3	N/A	93.4	86.7	43.9	N/A	0
Kim et al, 2019	8.2/4	N/A	N/A	N/A	N/A	N/A	N/A
Hu et al, 2020	4.0/20.8	1.5/9.6	N/A	N/A	N/A	N/A	N/A
Hori et al, 2020	4.6	N/A	N/A	N/A	20.3	N/A	N/A

N/A = Not available, OS = overall survival, PFS = progression-free survival, TACE = transarterial chemoembolization.

## 15. Agents used

All 28 studies mentioned agents that were used for performing TACE. Doxorubicin, incorporated in 15 studies,^[19–24,27–31,34,38,39,44]^ was the most used chemotherapeutic agent owing to its potent antitumor properties. This anthracycline derivative functions by intercalating DNA, impeding topoisomerase II activity, and disrupting nucleic acid synthesis, thus exhibiting robust cytotoxic effects against tumor cells. Cisplatin was mentioned in eleven studies,^[22,24–26,28,30,36,38,39,41,46]^ which operates as an alkylating agent inducing DNA cross-linking, triggering apoptosis in neoplastic cells. Mitomycin was used in 9 studies.^[19,22,28,31,32,35,41,42,46]^ It exerts its cytotoxicity by inhibiting DNA synthesis through alkylation, functioning as a potent antineoplastic agent. Epirubicin was used in 7 studies,^[32,35,36,41,42,45,46]^ it belongs to the anthracycline class, sharing similar mechanisms to Doxorubicin, and showcases efficacious antitumor properties. Adriamycin (an anthracycline antibiotic) in 4,^[33,37,40,43]^ and fluorouracil in 3 studies.^[33,43,46]^ Fluorouracil interferes with nucleic acid synthesis, disrupting RNA function, and impeding DNA synthesis. While the majority of the studies used a combination of chemotherapeutic agents, 7 studies used single agents for their intervention,^[20,21,25–27,29,34]^ highlighting the different variations of TACE across studies.

## 16. Discussion

The outcomes of TACE in advanced HCC (classes B and C) showcased variability across studies, however, favoring TACE in their conclusion. Our review amalgamated insights from a diverse array of studies, shedding light on the safety and efficacy of TACE in advanced HCC. Of safety assessments across nineteen studies, considerable attention was devoted to liver function tests and patient-reported symptoms, notably focusing on markers such as AST, ALT, and bilirubin levels, alongside reported manifestations including fever, pain, vomiting, and gastrointestinal complications. These collective observations underscore the inherent complexities and risks associated with TACE, necessitating comprehensive monitoring protocols post-procedure to mitigate potential adverse events.

The exception was seen in Hu et al, where patients on TACE were found to have shorter average survival (4 months) compared to hepatic arterial infusion chemotherapy.^[45]^ When an in-depth look was taken, it was revealed that the study undertook only major portal vein tumor thrombosis (PVTT) demographics in the group. According to the Liver Cancer Study Group of Japan, there are 4 macroscopic classifications of PVTT, of which Vp3 PVTT refers to the presence of PVTT in the first-order branches of the portal vein, and Vp4 PVTT refers to the presence of PVTT in the main trunk of the portal vein.^[47]^ While the specific reasoning is poorly understood, multiple previous studies demonstrated that for patients with major PVTT, TACE has been an unfit option, leading to an OS of 3 to 9.5 months, under ideal conditions.^[22,29,34,48]^

Apart from Hu et al, all other studies demonstrated better efficacy for TACE, making it a potentially safer choice. It is also imperative to note that the primary cause of mortality among HCC patients typically stems from intrahepatic tumor progression or liver failure rather than extrahepatic metastasis, hence managing intrahepatic tumors through locoregional therapies is more important.^[49]^ Hence the clear picture of TACE and its survival benefits can be appreciated clearly once local disease is halted.

The presented evidence suggests a potential role for TACE in specific subsets of advanced HCC patients with vascular invasion and favorable liver function. The study findings align with the notion that TACE could offer a survival benefit, especially when intrahepatic tumor control is crucial or when the tumor is PVTT 1 or 2.^[22,29,34,48]^

According to BCLC staging, atezolizumab-bevacizumab or durvalumab-tremelimumab are the first-line treatments for advanced HCC. However, due to their high cost, unaffordability and unavailability for many, it was imperative to find alternate solutions for advanced HCC.^[50]^

While there have been multiple previous papers suggesting a role of TACE in vascular invasion in HCC or extrahepatic metastasis,^[19,20,31,44,46,51]^ ours took into consideration the greatest number of studies to date, including studies without comparison groups, hence, paving the way for future research work. Moreover, our study included multiple variables to assess safety and efficacy outcomes, hence ensuring that comprehensive includes all particular aspects. Given the heterogeneous nature of HCC and the diverse treatment approaches employed in clinical practice, future studies could benefit from exploring the impact of TACE in combination with other locoregional or systemic therapies. Investigating multimodal treatment strategies, such as TACE combined with radiofrequency ablation or immunotherapy, may offer synergistic benefits and improve patient outcomes in select cohorts. Furthermore, incorporating patient-reported outcomes and healthcare utilization metrics into future studies could provide a comprehensive understanding of the holistic impact of TACE on patients’ lives and healthcare resource utilization.

This study has several limitations that warrant consideration. Firstly, the review encompassed numerous studies, revealing a striking disparity in outcomes regarding the efficacy of TACE in advanced HCC. This disparity challenges the universality of TACE’s success compared to conservative treatments, highlighting the need for caution in drawing definitive conclusions. Secondly, despite the inclusion of a substantial number of studies, our review did not incorporate a meta-analysis due to the unavailability of uniform data. This absence limits the depth of our synthesis and comprehensive understanding of the collective evidence, potentially influencing the certainty of our conclusions. Third, a notable observation was the diversity in treatment approaches across different regions. This variability could have influenced the reported outcomes and potentially restricted the applicability of our findings on a global scale. Additionally, certain studies lacked comprehensive patient demographic information, particularly regarding macroscopic classifications like PVTT. The omission of these critical details might have introduced biases in our interpretation of outcomes, particularly in patients with major PVTT. While minor, the post-TACE observed side effects, as documented in Table 1, underscore the significance of vigilant post-procedural monitoring. This emphasizes the need for meticulous reporting of adverse events in future studies and comprehensive patient care. Lastly, despite the promising findings suggesting a potential role for TACE in specific subsets of advanced HCC patients, our study may not encompass all variables or scenarios influencing treatment outcomes. There might exist unexplored factors that could impact the efficacy of TACE in different patient cohorts.

## 17. Conclusion

The systematic exploration of treatment outcomes, as evidenced by the diverse study data, offers crucial insights into the efficacy and safety of TACE in addressing HCC with vascular invasion or extrahepatic metastasis. Overall, TACE can be considered a safe alternative and a choice for advanced disease patients (both extrahepatic metastasis vascular invasion ones) and in those treated with TACE alone as demonstrated by outcomes in our results, when compared to other plausible options. This targeted approach holds promise in not only optimizing therapeutic benefits but also in minimizing associated risks, thereby enhancing the overall management and outcomes for this challenging patient population. However, the documented heterogeneity in outcomes emphasizes the necessity for further rigorous research.

## Author contributions

**Conceptualization:** Muhammad Usman Younas, Abdullah Saeed, Muhammad Ramzan, Muhammad Junaid Tahir, Khabab Abbasher Hussien Mohamed Ahmed, Ali Ahmed.

**Data curation:** Muhammad Usman Younas, Abdullah Saeed, Muhammad Ramzan, Khabab Abbasher Hussien Mohamed Ahmed, Ali Ahmed.

**Formal analysis:** Muhammad Usman Younas, Abdullah Saeed, Muhammad Ramzan, Ali Ahmed.

**Funding acquisition:** Muhammad Usman Younas, Abdullah Saeed, Muhammad Ramzan.

**Investigation:** Muhammad Usman Younas, Abdullah Saeed, Muhammad Ramzan, Ali Ahmed.

**Methodology:** Muhammad Usman Younas, Abdullah Saeed, Muhammad Ramzan, Muhammad Junaid Tahir, Ali Ahmed.

**Project administration:** Muhammad Usman Younas, Abdullah Saeed, Muhammad Ramzan, Muhammad Junaid Tahir.

**Resources:** Muhammad Usman Younas, Abdullah Saeed, Ali Ahmed.

**Software:** Muhammad Usman Younas, Abdullah Saeed, Ali Ahmed.

**Supervision:** Muhammad Usman Younas, Muhammad Junaid Tahir, Ali Ahmed.

**Validation:** Muhammad Usman Younas, Abdullah Saeed, Muhammad Ramzan, Muhammad Junaid Tahir, Khabab Abbasher Hussien Mohamed Ahmed, Ali Ahmed.

**Visualization:** Muhammad Usman Younas, Abdullah Saeed, Muhammad Ramzan, Muhammad Junaid Tahir, Khabab Abbasher Hussien Mohamed Ahmed, Ali Ahmed.

**Writing – original draft:** Muhammad Usman Younas, Abdullah Saeed, Muhammad Ramzan, Muhammad Junaid Tahir, Khabab Abbasher Hussien Mohamed Ahmed, Ali Ahmed.

**Writing – review & editing:** Muhammad Usman Younas, Abdullah Saeed, Muhammad Ramzan, Muhammad Junaid Tahir, Khabab Abbasher Hussien Mohamed Ahmed, Ali Ahmed.

## Supplementary Material


