# Supplementary material for: Transarterial chemoembolization in hepatocellular carcinoma: exploring its role in vascular invasion and extrahepatic metastasis: A systematic review
Source: Medicine (Baltimore). 2025 Feb 21;104(8):e41570. doi: 10.1097/MD.0000000000041570 (PMC11856889; doi:10.1097/MD.0000000000041570)
Supplement: Supplementary file 1 [file medi-104-e41570-s001.docx]

| **Platform** | **Search String** |
| --- | --- |
| PubMed | ("transarterial chemoembolization" [All fields] or "TACE" [All fields]) AND ("hepatocellular carcinoma" [All fields] or "HCC" [All fields] or "liver cancer" [All fields] or "liver tumor" [All fields]) AND ("portal vein invasion" [All fields] or "portal vein tumor thrombus" [All fields] or "PVTT" [All fields] or "vascular invasion "[All fields] or "extrahepatic spread" [All fields] or "metastatic" [All fields] or "metastasis" [All fields]) |
| Cochrane | ("transarterial chemoembolization" or "TACE") AND ("hepatocellular carcinoma" or "HCC" or "liver cancer" or "liver tumor") AND ("portal vein invasion" or "portal vein tumor thrombus" or "PVTT" or "vascular invasion " or "extrahepatic spread" or "metastatic" or "metastasis") |
| Google Scholar | ("transarterial chemoembolization" or "TACE") AND ("hepatocellular carcinoma" or "HCC" or "liver cancer" or "liver tumor") AND ("portal vein invasion" or "portal vein tumor thrombus" or "PVTT" or "vascular invasion " or "extrahepatic spread" or "metastatic" or "metastasis") |

**Supplementary Table 1: Platforms and the search string used**
